# Trends in the histopathology of childhood nephrotic syndrome in Ibadan Nigeria: preponderance of idiopathic focal segmental glomerulosclerosis

**DOI:** 10.1186/s12882-015-0208-0

**Published:** 2015-12-15

**Authors:** Adanze O. Asinobi, Adebowale D. Ademola, Clement A. Okolo, Joseph O. Yaria

**Affiliations:** Department of Paediatrics, College of Medicine, University of Ibadan, Ibadan, Oyo State Nigeria; Department of Paediatrics, University College Hospital Ibadan, Ibadan, Oyo State Nigeria; Department of Pathology, College of Medicine, University of Ibadan, Ibadan, Oyo State Nigeria; Department of Pathology, University College Hospital Ibadan, Ibadan, Oyo State Nigeria; Department of Medicine, University College Hospital Ibadan, Ibadan, Oyo State Nigeria

**Keywords:** Renal histopathology, Childhood nephrotic syndrome, FSGS, MPGN, MCD, Ibadan Nigeria

## Abstract

**Background:**

Reports on the histopathology of childhood nephrotic syndrome (NS) had emanated from our Centre since the 1960s and by the late 1980s and early 1990s, a change was observed and reported. Taking into consideration the worldwide changing trend in the histopathology of the NS and our Unit policy change in the indications for renal biopsy, a change was envisaged. We therefore evaluated the current histologic pattern of childhood NS in Ibadan with the view to highlighting any variations from the past and comparing the findings with regional and global trends.

**Methodology:**

We reviewed our database and analyzed the renal biopsy findings in patients who were biopsied before treatment was administered between 1997 and 2001 and those with mostly idiopathic steroid resistant NS (SRNS) and secondary NS, managed between 2006 and 2013. A comparative analysis of the findings from the present study was carried out with two previous reports from our Unit in the 1970s and early 1990s and also with reports from other Centres.

**Results:**

A total of 78 patients had successful biopsies done during the study period in children aged between 2 ½ and 16 years. In both pre-treatment biopsy era (1997–2001) and post-treatment biopsy era (2006–2013), focal segmental glomerulosclerosis (FSGS) predominated. 75 % of the patients had idiopathic NS and among the patients that had idiopathic steroid resistant NS, FSGS was the most common followed by MPGN. For secondary NS, MCD was the most common but could be the early stages of either membranous nephropathy (MN) or FSGS. Chronic pyelonephritis and chronic interstitial nephritis occurred in 25 % of the study population but they were more prevalent in secondary nephrotic syndrome.

**Conclusion:**

FSGS is the most common histopathology in children requiring renal biopsy in Ibadan presently. FSGS is also the most common histopathology in idiopathic SRNS, which is in keeping with reports from most parts of the world.

There has been a transition from the preponderance of Quartan Malarial Nephropathy (QMN) in the 1960s to MPGN in the 1980s to FSGS presently. This has great implications with regards to searching for new aetiologic factors, providing more efficacious treatment modalities and ensuring facilities for immunofluorescence, electron microscopic and genetic studies.

## Background

The variations between the preponderant histologic patterns in children with Nephrotic Syndrome (NS) in the tropics and their response to therapy as distinct from those of the temperate countries were well documented in our setting several decades ago [1–5]. The clinico-pathologic entity, Quartan Malarial Nephropathy (QMN) was predominant in Ibadan in the 1960s when it was seen in 81 % of the patients [4]. In the 1980s, Abdurrahman et al. working in Kaduna, Northern Nigeria reported QMN in 20 % of the biopsy specimens they studied [6, 7]. In the late 1980s and early 1990s, studies done in our Centre showed a preponderance of Membrano-Proliferative Glomerulonephritis (MPGN) [8]. In South-west Nigeria where our Centre is located, a rarity of Minimal Change Disease (MCD), the predominant lesion seen among Caucasian children, has also been well demonstrated in both children and adults [4, 8, 9] and could have been responsible for the high prevalence of steroid resistance in our environment. However, in a multi-ethnic, multi-cultural and hugely populated country, like Nigeria, with people of varying socio-economic backgrounds, variations in steroid sensitivity and histological patterns have been demonstrated between regions [10–15].

Apart from environmental factors, genetic factors may play a role in the histologic pattern seen in this condition. In multi-racial countries for instance, people of African descent have been shown to be more at risk for developing Steroid Resistant Nephrotic Syndrome (SRNS), especially from Focal Segmental Glomerulosclerosis (FSGS) [16–19]. Surprisingly, from the 1990s, an increasing prevalence of FSGS in children [20–26] and adults [27, 28], has been reported in several continents of the world. The changing institutional indications for renal biopsy in different parts of the world could not fully explain this trend and suggested reasons include environmental pollution and morbid obesity [29, 30].

Before 2006, the Unit policy was to biopsy all NS patients that presented in our Unit who had no contraindications before treatment. This was predicated on the rarity of MCD and high level of steroid toxicity seen in children managed at our Centre [3, 5]. The subsequent findings and recommendation to withhold steroids from African children with structural abnormalities by workers in South Africa gave more credence to that practice [16]. However, consequent on some observed epidemiological changes, especially with regards to increasing response of our children to steroid therapy [12] and the fact that steroid responsiveness is considered a stronger determinant of prognosis in childhood NS than the histologic pattern [31–33], our patients now get treated with steroids before biopsy. The indications for renal biopsy at our Centre are now more in line with global practices i.e. steroid resistance, secondary NS and atypical presentations. Taking into consideration the world-wide changing trend in the histopathology of the NS and our unit policy change in the indications for renal biopsy, a change was envisaged.

### Objective

To evaluate the current histologic pattern of childhood NS in Ibadan, and highlight any variations from the past and compare findings with regional and global trends.

## Patients and methods

### Study location

The University College Hospital (UCH), Ibadan is the first and largest tertiary care centre in Nigeria. The hospital is located in Ibadan, the capital city of Oyo State, in the South-Western region of Nigeria. The Paediatric Nephrology Unit of the Hospital was established since the 1960s.

### Study design

This is an on-going descriptive study on histopathological results of renal biopsy specimens obtained from our Unit. A Biopsy register is kept in the Histopathology Unit alongside records in the Paediatric Nephrology Unit.

### Study population

Between 1997 and 2001, in accordance with the Unit protocol, all consecutive NS patients aged 16 years and below who had no contraindication were planned for biopsy before definitive treatment, in the absence of logistic hindrances (this period is referred to as the pre-treatment biopsy era). Between 2006 and 2013, following a change in the unit protocol, NS patients who failed to go into remission following the administration of oral prednisolone at 60 mg/m^2^/day for at least 4 weeks in divided doses (i.e. steroid resistant NS patients), steroid-dependent patients (relapsed as steroids were being tailed off) and frequent relapsers (had ≥2 relapses in 6 months or >3 relapses in I year) requiring second-line drug treatment were biopsied. In addition, those with secondary NS and atypical presentation (macroscopic haematuria, azotaemia and hypertension, intermittent massive proteinuria) were also biopsied. The period from 2006 to 2013 will subsequently be referred to as Post-treatment biopsy era. A written informed consent was obtained from each patient’s parent or guardian and assent was also obtained from children aged seven years and above pre-biopsy.

### Patients information

Patient’s demography, clinical presentations, diagnostic tests and results were recorded. Clinical and laboratory data that led to the establishment of the diagnosis of the NS were ensured i.e. a combination of massive proteinuria (proteinuria of 3+ and above on dipstick urinalysis with a 24-h urinary protein of >40 mg/m^2^/h), hypo-albuminaemia (serum albumin of <2.5 g/dl), hyperlipidemia (serum cholesterol >220 mg/dl) and oedema.

Investigations routinely carried out on these patients were dipstick urinalysis, urine microscopy/culture/sensitivity, 24-h urinary protein estimation, spot urine protein/creatinine ratio estimation, creatinine clearance, lipid profile, serum electrolytes, urea and creatinine, full blood count, haemoglobin electrophoresis and blood film for malaria parasites. Others were Hepatitis B & C screening, HIV screening, ASO titre estimation and abdominal ultrasonography.

For renal biopsies, normal coagulation profile and absence of UTI were ensured. Blood grouping and cross-matching were done and a unit of blood was routinely reserved for the biopsy. All the renal biopsies performed from 2006 were carried out under real-time ultrasound guidance using 16-18G spring-loaded semiautomatic biopsy needles after adequate sedation and application of local anesthetics. Before then, the regular ‘Tru-cut’ disposable needle (Travenol) was used semi-blind i.e. unguided biopsy after ultra-sound determination of the number, the location, the dimensions and depth of the kidneys had been carried out and a surface marking done. Two kidney biopsy cores were taken from each patient for light microscopy.

For light microscopy, samples were fixed in formalin, embedded in paraffin wax and sections cut to 4 μ thickness. The sections were stained with haematoxylin and eosin (H&E), Periodic Acid Schiff (PAS) and Jane’s methenamine silver. Masson Trichrome and Congo-red stains were used whenever required. All specimens were studied by an experienced renal histopathologist (OCA) and his team. Some of the specimens were discussed at our Clinico-pathologic meetings. Immunofluorescence studies and electron microscopy were not carried out because facilities for these are presently not available for diagnostic purposes in Nigeria. The indications for kidney biopsy and biopsy reports were all noted and inputted into a proforma and then to an Excel spread sheet.

### Data analysis

Statistical analysis was carried out using STATA version 12.0. Simple descriptive statistics such as mean ± SD were used for continuous variables. Percentages were used for categorical data. The results were analyzed for their statistical significance using Mann Whitney U and Kruskal Wallis test for continuous variables, chi-square test was used for discrete variables. *P-*value <0.05 was considered significant.

### Ethical statement

An ethical approval for this study was obtained from the University of Ibadan/University College Hospital, Ibadan Joint Ethical Committee. The study analyzed the outcome of our routine work in the Paediatric Nephrology Unit and patients’ individual data are not traceable to them.

## Results

Fifty-eight children were diagnosed with NS in the pre-treatment era while 106 children were treated in the post-treatment era. Seventy-eight (78) patients had successful biopsies done during the period of study. Of the 56 patients biopsied between 2006 and 2013 (Post-treatment biopsy era), the major indication was SRNS in 30 patients (53.6 %); followed by secondary NS (25 %), atypical features (10.7 %) and other steroid-related issues (10.7 %).

Mean age in the pre-treatment biopsy era (1997–2001) was 8.9 ± 4.0 years and 8.3 ± 3.7 years in the post treatment biopsy era. The children in the pre-treatment era were predominantly female (59.1 %) as opposed to 33.9 % in the post-treatment era (p = 0.041).

**Fig. 1:**
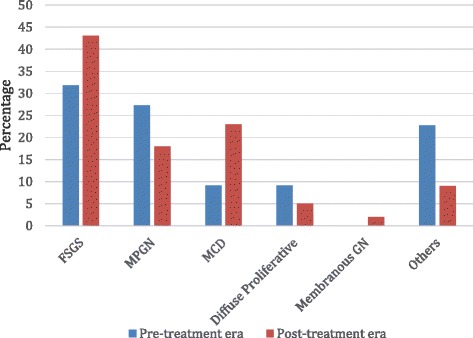
Overall histologic pattern in the present study

**Fig. 2:**
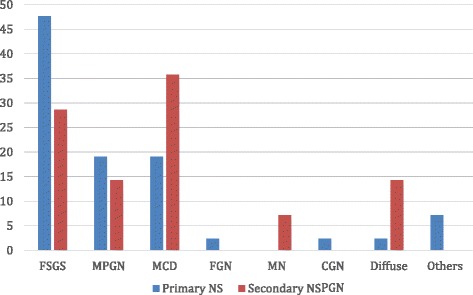
Distribution of Primary and Secondary cases in the Post-steroid treatment era (2006–2013)

**Fig. 3:**
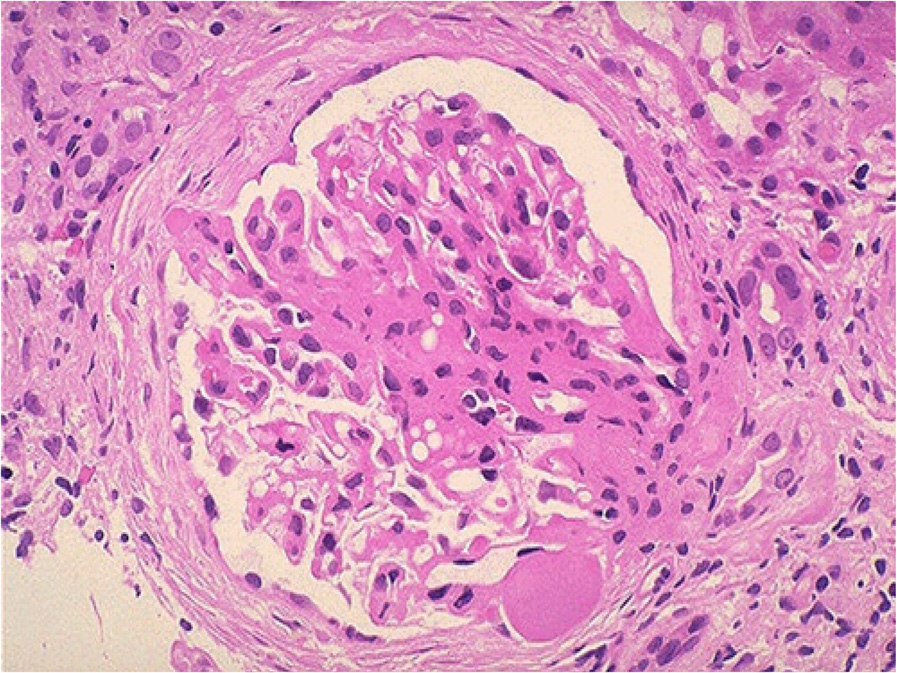
This is a photomicrograph of focal segmental glomerulosclerosis (FSGS). An area of collagenous sclerosis runs across the middle of this glomerulus

Figure 1 gives an indication of the various histological diagnoses from each treatment era while Fig. 2 shows the proportions of Primary and Secondary NS in the post-treatment era. For the pre-treatment era, FSGS predominated (31.8 %), followed closely by MPGN in 27.3 %. MCD and Diffuse Proliferative Glomerulonephritis (DPGN) were seen in 9.1 % each and other histological types in 22.7 %. In the post-treatment era, FSGS was seen in 43 %, MPGN in 18 %, MCD in 23 % and 16 % were for others such as FGN, DPGN, membranous nephropathy (MN), Chronic GN, ESKD, HIVAN and Crescentic GN. Overall, FSGS (39.8 %) was the most prevalent followed by MPGN (20.5 %) and thirdly MCD (19.2 %). Figure 3 shows the micrograph of one of our patients with FSGS.

With regards to primary NS in the post-treatment era, FSGS (47.6 %) was the predominant histology type while MPGN and MCD were seen in 19 % each. However among the secondary cases, MCD (35.7 %) predominated followed by FSGS (28.6 %) and MPGN (14.3 %) following (Fig. 2). Interestingly, 4 out of the 5 cases of MCD were Hepatitis B surface antigen positive and one had Graves’ disease. The 4 cases of FSGS had Sickle Cell disease (1), HIV (1) and 2 were Hepatitis B surface antigen positive. Two cases of MPGN seen were Hepatitis B and Tuberculosis related. The only case of MN seen had HIV. It is note-worthy that none of these patients had a positive blood film for Plasmodium malariae, Schistosoma mansoni ova in stool nor Schistosoma haematobium ova in urine.

Among the 30 patients that had primary (1°) SRNS, 60 % had FSGS, 27 % had MPGN, 3 % had MCD while the others accounted for 10 % (Table 1). Table 1 also shows the comparison between the histological pattern of 1° SRNS from the present study with some local and international studies.

**Table 1:** Comparison of idiopathic steroid resistant nephrotic syndrome

	Bonilla-Felix et al 1999 [23]	Gulati et al. 2006 [34]	Seif et al. 2013 [51]	Mubarak et al. 2012 [42]	Olowu et al. 2010 [14]	Obiagwu et al. 2014 [39]	Present study
Location	USA	India	Egypt	Pakistan	Ile Ife	Kano	Ibadan
Period of Study	1987–1997	1990–1996	2005–2011	2009–2011	2001–2007	-	2006–2013
No. of Subjects	152	33	53	147	19	11	30
Mean age in years	5.3	5.7	6.7	7	8	8	9
MPGN	5.0 %	1.6 %	7.5 %	4.8 %	43.5 %	9.1 %	27 %
FSGS	31 %	58.8 %	30.2 %	38.7 %	39.1 %	54.5 %	60 %
MCN	35 %	17.6 %	24.5 %	23.1 %	4.35 %	27.3 %	3 %
MesPGN	25 %	17.6 %	1.9 %	10.2 %	8.7 %	-	-

Concerning the patients with MCD, five out of the thirteen were secondary, and for the primary cases their characteristics were as follows: two were late responders; one was a frequent relapser; one was steroid dependent; one was steroid resistant and three had atypical features. Three out of the 30 1° SRNS patients had MCD (10 %) but 2 of them proved to be late responders, only 1 patient (3.3 %) had true steroid resistance.

As regards associations, it was packed cell volume (PCV) only that showed significant association with histological types. Patients with FSGS had lower hematocrit levels compared to the other common histological types – MPGN and MCD (P = 0.01). Creatinine levels although more elevated in children with FSGS failed to reach statistical significance (p = 0.05) (Table 2). Table 3 compares the morphologic patterns of this study with others from other centres.

**Table 2:** Association of Clinical and Laboratory Features with Histologic Types

	FSGS	MPGN	MCD	OTHERS	P
AGE (YEARS)	9.5 (3.8)	8.7 (3.4)	7.5 (3.2)	9.8 (3.0)	0.37
*PCV (%)	28.1 (7.3)	34.6 (5.0)	38.3 (4.1)	27.2 (10.5)	0.01
CR (MG/DL)	2.0 (1.4)	0.9 (0.4)	0.7 (0.4)	3.7 (4.1)	0.05
CHOL (MG/DL)	416.1 (165.9)	329.9 (147.7)	405.2 (246.7)	437.0 (162.6)	0.77
TG (MG/DL)	302.5 (171.8)	260.0 (233.0)	276.4 (154.3)	300.0 (135.4)	0.97
HDL (MG/DL)	50.7 (14.8)	47.3 (10.7)	51.0 (17.3)	60.3 (40.9)	0.82
LDL (MG/DL)	278.8 (170.2)	187.8 (96.3)	277.0 (199.0)	292.6 (115.0)	0.62
DURATION IN WEEKS (MEDIAN, IQR)	2 (1–4)	2 (1–4)	8 (2–48)	3 (2–8)	0.744

*PCV - The only association of significance

**Table 3:** Comparison with other studies in our environment and globally

	Hendrickse (1972) [4]*a*	ISKDC Study (1978) [52]*b*	Abdurrahman et al. (1990) [6]*c*	Asinobi et al. (1999) [8]*d*	Present study *e*
Location	Ibadan	Multinational	Zaria	Ibadan	Ibadan
Number	63	521	99	41	78
Mean age (year)	No data	-	5.8	7.9	9.5
Quartan malarial nephropathy	51 (81.0)	-	20 (20.4)	-	_
Mes.PGN	5 (7.9)	(12) 2.3	-	-	-
MPGN	-	(39) 7.5	25 (25.5)	21 (51.2)	16 (20.5)
MCD	5 (7.9)	(398) 76.4	9 (9.2)	4 (9.8)	15 (19.2)
Membranous	-	(8) 1.5	2 (2.0)	4 (9.8)	1 (1.3)
Proliferative GN	-	-	19 (19.4)	2 (4.9)	8 (10.3)
Chronic GN	-	-	-	1 (2.4)	3 (3.8)
FSGS	1 (1.6)	(36) 6.9	2 (2.0)	2 (4.9)	31 (39.8)
Others	1 (1.6)	(28) 5.4	21 (21.5)	7 (17.0)	4 (5.1)
Total	63 (100)	521 (100)	99 (100)	41 (100)	78 (100)

a,b,c,d referenced studies above were Pre-treatment biopsies while e, was majorly Post-treatment

A 25 % prevalence of chronic pyelonephritis and chronic interstitial nephritis was seen in this study population and these findings were more prevalent in secondary nephrotic syndrome.

## Discussion

This study has shown a preponderance of FSGS in the NS patients who had renal biopsies in our Unit both in the pre-treatment and post-treatment biopsy eras. It should be noted that not all children that required biopsies had them because of financial constraints and lack of appropriate facilities. The predominating histologic patterns after FSGS were MPGN and MCD. For the patients biopsied, in the post-treatment era, the major indications were steroid resistance, secondary NS and atypical presentation. In 75 % of cases, no cause could be identified.

Although it is generally accepted that the most important prognostic indicator in childhood NS is steroid responsiveness, the underlying histopathology is also very important in prognostication, especially where patients are steroid resistant [34]. The major indication for the biopsy in the post-treatment era in this study was steroid resistance in over 50 % of the patients. In interpreting and comparing histologic reports from different centres, the indications for renal biopsy, steroid exposure, the time periods of the study which have been shown to produce varying results over decades in the same location [20, 26, 35] need to be critically considered. Other important considerations are patient population in terms of age [36–38], race and whether native or transplant kidneys are studied. All the patients studied were black Nigerians and the kidneys were all native kidneys.

One of the notable findings from this study is that FSGS is the predominant histology in our patients with steroid resistant NS. From reports of studies carried out in our locale, although the cohorts are not large [14, 39] and various other parts of the world, FSGS either ranks first or second as the most common cause of idiopathic steroid resistant NS [40–42].

Another key observation in this study is the changing trend of the preponderant histopathology of childhood NS in our Centre from QMN which predominated from the 1960s [4] to early 1980s when focal sclerosis was seen in only 1 % of the cases, to MPGN from the late 1980s to the mid-1990s [8] when the prevalence of FSGS rose to 4.9 % and now FSGS predominates. Even in the pre-treatment biopsies carried out between 1997 and 2001, MPGN was slightly overtaken by FSGS. The major explanation that can be adduced for this observation is a change in the aetiologic factor(s) of childhood NS in Ibadan. It is expedient that new aetiologic factors be sought vigorously.

Surprisingly, the proportion of MCD patients increased in this cohort of patients but a significant proportion was secondary. 25 % of the patients had some identifiable co-morbidities namely Hepatitis B infection, HIV and SCD. MCD was the most common histopathology in that group followed by FSGS. Minimal change histology was seen in 4/5 patients with Hepatitis B infection, which is unusual. We postulate that the light microscopy result seen in these patients may be early stages of membranous nephropathy, which is more associated with Hepatitis B infection. Repeat renal biopsies with immunofluorescence and electron microscopic studies should be indicated in these patients especially in those that were seropositive for HBsAg.

The high proportion of FSGS in our setting may explain the high incidence of steroid resistance among children and adolescents with NS. With regards to the specific types of FSGS, these patients who were majorly steroid-resistant showed the “Not-otherwise specified FSGS” and this was not surprising. It is envisaged that patients with the ‘’tip lesion’ type would have responded to steroid therapy. Only a case of collapsing FSGS was seen. We did not encounter many children with HIV requiring renal biopsy because as soon as the highly active anti-retroviral therapy (HAART), the proteinuria resolved. Out of the five patients with HIV and massive proteinuria seen during the latter years, two were biopsied; one had HIVAN and the other Membranous nephropathy. Other renal diseases encountered were AKI, CKD but not many with the NS as documented in our previous study [43].

With the change in the indications for kidney biopsy and the observed increase in the proportion of our patients being steroid responsive [12] we cannot dispute the fact that there is a real increase in the proportion of our nephrotic children who have MCD. The patients with MCD were predominantly steroid sensitive, but were either late responders, frequent relapsers or steroid dependent. Only one patient had true steroid resistance. One of the patients that had primary MCD had associated interstitial nephritis, which probably led to the atypical response. This stresses the fact that the majority of Nigerian children who have minimal change disease are steroid sensitive.

One of the noteworthy findings is that 25 % of these patients had chronic interstitial nephritis and chronic pyelonephritis. These are conditions that could singlehandedly lead to chronic kidney disease. In over 50 % of our patients, herbal remedies were utilized but only after the onset of their symptoms. The use of these toxic agents in the background of severe kidney injury manifesting as nephrotic syndrome may account for the atypical manifestations and incomplete remission to steroid therapy we see in these patients. The finding of chronic pyelonephritis corroborates the findings of Ibadin MO who reported a high prevalence of UTI in children with NS in Benin, Nigeria and this could further damage the kidneys [44].

When all patients who were untreated were put together, MPGN was just slightly exceeded by FSGS. It is possible that a higher proportion of the patients with MPGN responded to steroids than FSGS patients and therefore did not require renal biopsy. Overall proliferative lesions were frequently seen, probably pointing to infectious agents, though unidentified, still contributing to the development of NS in our setting. Membranous nephropathy remained rare in Ibadan and in this study it was not associated with Hepatitis but with HIV infection. It is gratifying to note that vaccination against Hepatitis B, about the commonest viral infection associated with this condition presently, is now available nationwide and very soon, Hepatitis B associated NS will not pose a big challenge. It is possible that other infectious agents such as the PARVOVIRUS that has been associated with CKD, Aplastic anaemia in Sickle cell anaemia patients may be an important aetiologic agent in our setting.

The recent discovery that two sequence variants in APOL1 (G1: rs73885319, G2: rs71785313) are more common in individuals of African descent (Yorubas of South Western Nigeria) compared with Europeans and that the disease associated alleles are more common in African Americans (AA) with FSGS compared with AA with no disease, may be important in our setting [45–47]. This is more so as most of our patients are of the Yoruba tribe. Recent reports in adult Nigerian populations have demonstrated APOL1 risk variants’ association with non-diabetic forms of CKD among Nigerians of Yoruba ethnicity in South-west Nigeria and also among the Igbos in South-east Nigeria [48, 49].

The concept of podocytopathies as the unifying hypothesis for glomerular diseases [50] is presently widely propagated especially in relation to FSGS and MCNS. The complex interaction between environmental and genetic factors may then result in podocyte injuries of varying degrees. We hypothesize therefore that infectious agents acting on the genetically predisposed, probably those with APOL1 genetic mutation, may be responsible for the preponderance of FSGS seen in Ibadan, which is a Yoruba Land.

Age at initial presentation has been shown to be an important factor on the disease distribution frequency in childhood nephrotic syndrome. The report of the International Study of Kidney disease in Children [40] showed that 70 % of children with MCD are younger than 5 years while Baqi et al. showed that only 20–30 % of adolescent nephrotic patients have MCNS [37]. FSGS develops in children at a median age of 6 years among Caucasians [40]. Previous workers have shown that the probability of having FSGS or MPGN as the underlying cause of NS increases with increasing age, whereas the risk of having MCNS is inversely related to the age at presentation with nephrotic syndrome. When compared with reports from Caucasian children and the reports from certain parts of our country where high steroid sensitivity has been demonstrated [13, 15], our patients requiring renal biopsy were older. More than 60 % of the patients with 1° steroid resistant FSGS were aged 8 years and above in the present study.

The treatment of steroid-resistant FSGS poses a big challenge to paediatric nephrologists and is worse for those working in resource-limited settings like ours. These patients if not aggressively treated are likely to end in ESKD. The more potent second and third-line immunosuppressives should be the ideal drugs to be administered to these patients but they are unaffordable by them. Presently, the second-line drugs viz cyclosporine, Mycophenolate mofetil are more accessible in our environment but are still not affordable by any of our patients. There is an urgent need to make available appropriate medications that will prevent progression to ESKD and to intensify efforts at discovering the aetiology of this condition and its prevention.

## Conclusions

Going by the current indications for renal biopsy in nephrotic children in Ibadan, the most common histopathological diagnoses one is likely to encounter are FSGS, MPGN and MCD. FSGS predominated in the total population studied and was clearly the predominant histology in the SRNS patients. There has been a transition from the preponderance of Quartan Malarial Nephropathy (QMN) in the 1960s to MPGN in the 1980s to FSGS presently. This has great implications with regards to searching for new aetiologic factors, providing more efficacious treatment modalities and ensuring facilities for immunofluorescence and electron microscopic studies. Even though light microscopy has assisted us in describing the histopathology of childhood NS which is helpful with prognostication, there is a great need for immunofluorescence and electron microscopic studies to be re-instituted in our practice to further describe the possible aetiology of this condition. There is also an urgent need for genetic and molecular studies in these children to get a broader picture of this challenging condition in South-west Nigeria.

## Abbreviations

APOL 1: apolipoprotein L1
ASO titre: anti-streptolysin o titre
CKD: chronic kidney disease
DPGN: diffuse proliferative glomerulonephritis
ESKD: end stage kidney disease
FGN: focal glomerulonephritis
FSGS: focal segmental glomerulosclerosis
GN: glomerulonephritis
HIV: human immunodefiency virus
HIVAN: human immunodefiency virus associated nephropathy
iSRNS: idiopathic steroid resistant nephrotic syndrome
MCD: minimal change disease
MN: membranous nephropathy
MPGN: membranoproliferative glomerulonephritis
NS: nephrotic syndrome
PAS: periodic acid schiff
PCV: packed cell volume
QMN: quartan malarial nephropathy
SCD: sickle cell disease
SD: standard deviation
SRNS: steroid resistant nephrotic syndrome
UCH: University College Hospital
UTI: urinary tract infection
